# A Novel Single Cell RNA-seq Analysis of Non-Myeloid Circulating Cells in Late Sepsis****

**DOI:** 10.3389/fimmu.2021.696536

**Published:** 2021-08-16

**Authors:** Dijoia B. Darden, Xiaoru Dong, Maigan A. Brusko, Lauren Kelly, Brittany Fenner, Jaimar C. Rincon, Marvin L. Dirain, Ricardo Ungaro, Dina C. Nacionales, Marie Gauthier, Michael Kladde, Todd M. Brusko, Azra Bihorac, Frederick A. Moore, Tyler Loftus, Rhonda Bacher, Lyle L. Moldawer, Alicia M. Mohr, Philip A. Efron

**Affiliations:** ^1^Department of Surgery, University of Florida College of Medicine, Gainesville, FL, United States; ^2^Department of Biomedical Engineering, University of Florida College of Medicine, Gainesville, FL, United States; ^3^Department of Pathology, Immunology and Laboratory Medicine, University of Florida College of Medicine, Gainesville, FL, United States; ^4^Department of Biochemistry and Molecular Biology, University of Florida College of Medicine, Gainesville, FL, United States; ^5^Department of Medicine, University of Florida College of Medicine, Gainesville, FL, United States; ^6^Department of Biostatistics, University of Florida, Gainesville, FL, United States

**Keywords:** lymphocytes, immune cells, sepsis, transcriptome, scRNA-seq, chronic critical illness

## Abstract

**Background:**

With the successful implementation of the Surviving Sepsis Campaign guidelines, post-sepsis in-hospital mortality to sepsis continues to decrease. Those who acutely survive surgical sepsis will either rapidly recover or develop a chronic critical illness (CCI). CCI is associated with adverse long-term outcomes and 1-year mortality. Although the pathobiology of CCI remains undefined, emerging evidence suggests a post-sepsis state of pathologic myeloid activation, inducing suboptimal lymphopoiesis and erythropoiesis, as well as downstream leukocyte dysfunction. Our goal was to use single-cell RNA sequencing (scRNA-seq) to perform a detailed transcriptomic analysis of lymphoid-derived leukocytes to better understand the pathology of late sepsis.

**Methods:**

A mixture of whole blood myeloid-enriched and Ficoll-enriched peripheral blood mononuclear cells from four late septic patients (post-sepsis day 14-21) and five healthy subjects underwent Cellular Indexing of Transcriptomes and Epitopes by Sequencing (CITE-seq).

**Results:**

We identified unique transcriptomic patterns for multiple circulating immune cell subtypes, including B- and CD4^+^, CD8^+^, activated CD4^+^ and activated CD8^+^ T-lymphocytes, as well as natural killer (NK), NKT, and plasmacytoid dendritic cells in late sepsis patients. Analysis demonstrated that the circulating lymphoid cells maintained a transcriptome reflecting immunosuppression and low-grade inflammation. We also identified transcriptomic differences between patients with bacterial *versus* fungal sepsis, such as greater expression of cytotoxic genes among CD8^+^ T-lymphocytes in late bacterial sepsis.

**Conclusion:**

Circulating non-myeloid cells display a unique transcriptomic pattern late after sepsis. Non-myeloid leukocytes in particular reveal a host endotype of inflammation, immunosuppression, and dysfunction, suggesting a role for precision medicine-guided immunomodulatory therapy.

## Introduction

Successful implementation of guidelines from the Surviving Sepsis Campaign has led to a significant decrease in early mortality after sepsis (1, 2). Thus, three clinical trajectories or phenotypes are now present subsequent to surgical sepsis: early death (approximately 4%), rapid recovery (approximately 63%) and development of chronic critical illness (CCI; approximately 33%) (3). Specifically, CCI has been defined as patients who have prolonged ICU stays with unresolved organ dysfunction (4–6). CCI patients account for increased hospital costs and use of resources (3). Importantly, approximately 40% of CCI patients have poor 1-year outcomes with a worse 1-year quality of life and mortality after sepsis (3, 4, 7).

Although the underlying pathobiology of the CCI phenotype remains unclear, it is understood that CCI can result from a Persistent Inflammation, Immunosuppression, and Catabolism Syndrome (PICS) endotype (8–12). Previous studies indicate that the dismal long-term outcomes experienced by CCI patients are due in part to a failure of the host to return to their pre-sepsis immune status (5, 6, 9, 11, 13). Understanding the pathobiology of leukocytes in late sepsis, as well as the dysfunctional hematopoiesis that leads to this immune dyscrasia, will be vital to any successful immunomodulation of sepsis survivors. Our laboratory has previously published a pilot study specifically evaluating myeloid-derived suppressor cells in surgical sepsis survivors (14). However, we realized that studies on non-myeloid immune cell subtypes chronically after sepsis are lacking. Using single-cell RNA sequencing (scRNA-seq) in an increased number of samples, we sought to perform a novel transcriptomic analysis of the immune and non-immune subsets of non-myeloid circulating cell types, all of which contribute to the late sepsis survivor patient phenotype.

## Materials and Methods

### Study Design, Patient Enrollment, and Classification

The study was registered with clinicaltrials.gov (NCT02276417) and conducted by the Sepsis and Critical Illness Research Center at the University of Florida College of Medicine. All patients eligible for inclusion in the study were enrolled within 12 hours of initiating sepsis treatment. We used a delayed consent process, as approved by the Institutional Review Board. If written informed consent could not be obtained from the patient or their legally assigned representative within 96 hours of study enrollment, the patient was removed from the study and all collected biologic samples and clinical data were destroyed. Screening for sepsis was performed using the Modified Early Warning Signs-Sepsis Recognition System (MEWS-SRS) (13), which quantifies derangements in vital signs, white blood cell count, and mental status. All patients with sepsis were managed using a standardized, evidence-based protocol that emphasizes early goal-directed fluid resuscitation as well as other time-sensitive interventions such as administration of broad-spectrum antibiotics. Empiric antibiotics were chosen based on hospital antibiograms in conjunction with the suspected source of infection. Antimicrobial therapy was then narrowed based on culture and sensitivity data.

Inclusion criteria consisted of the following: (a) admission to the surgical or trauma ICU; (b) age ≥18 years; (c) clinical diagnosis of sepsis or septic shock as defined by the 2016 SCCM/ESICM International Sepsis Definitions Conference (Sepsis-3) (15) with this being the patient’s first septic episode; and, (d) entrance into our sepsis clinical management protocol as previously described (16). Exclusion criteria consisted of: (a) refractory shock (i.e. patients expected to die within the first 24 hours); (b) an inability to achieve source control (i.e. irreversible disease states such as unresectable dead bowel); (c) pre-sepsis expected lifespan <3 months; (d) patient/family not committed to aggressive management; (e) severe CHF (NYHA Class IV); (f) Child-Pugh Class C liver disease or pre-liver transplant; (g) known HIV with CD4^+^ count <200 cells/mm3; (h) organ transplant recipient or use of chronic corticosteroids or immunosuppressive agents; (i) pregnancy; (j) institutionalized patients; (k) chemotherapy or radiotherapy within 30 days; (l) severe traumatic brain injury (i.e. evidence of neurological injury on CT scan and a GCS <8); (m) spinal cord injury resulting in permanent sensory and/or motor deficits; or, (n) inability to obtain informed consent.

CCI was defined as an ICU length of stay greater than or equal to 14 days with evidence of persistent organ dysfunction, measured using components of the Sequential Organ Failure Assessment (SOFA) score (i.e. cardiovascular SOFA ≥ 1, or score in any other organ system ≥ 2) (3, 5, 12, 17). Patients with an ICU length of stay less than 14 days would also qualify for CCI if they were discharged to another hospital, a long-term acute care facility, or to hospice and demonstrated continuing evidence of organ dysfunction at the time of discharge. Those patients experiencing death within 14 days of sepsis onset were excluded from the analyses. Patients who neither died within 14 days nor developed CCI were defined as having a rapid recovery.

### Human Blood Collection and Sample Preparation

Ethylenediaminetetraacetic acid (EDTA)-anticoagulated human whole blood samples were collected by venipuncture from four patients in late sepsis (day 14-21) meeting Sepsis-3 criteria (15) and five healthy control subjects. Samples were stored on ice and processed within six hours after blood draw. Each sample was divided to undergo two separate enrichment processes. Peripheral blood mononuclear cells (PBMC) from half of each human whole blood sample were collected using Ficoll-Paque™ PLUS (GE Healthcare, Chicago, IL) and density gradient centrifugation. Myeloid cells were collected from the other half of each whole blood sample using RosetteSep™ HLA Myeloid Cell Enrichment Kit (Stemcell Technologies, Cambridge, MA). A 1:3 mixture of enriched PBMCs to myeloid cells from the four sepsis patients and five healthy control subjects underwent further analysis. Although single-cell technology allows for detection of smaller cell populations, the original samples were enriched to ensure our ability to adequately analyze and compare the small target population of MDSCs (especially in healthy controls, who have small populations of MDSCs), while also allowing us to characterize other important circulating immune cells present in late sepsis (e.g. lymphocytes).

### scRNA-seq/CITE-seq and Library Construction

Gene expression libraries were prepared from 5,000 cells using the Chromium Single Cell 5’ Bead and Library Kit v1 (10x Genomics). Libraries were sequenced on an Illumina HiSeq™ instrument at a target read depth of 50,000 reads per cell. Myeloid cells were labeled with oligo-tagged antibodies to CD33, CD11b, CD14, CD15, CD66b, Lox1 and HLA-DR. Labeled cells (5,000) were encapsulated for droplet-based CITE-seq utilizing the 10x Genomics Chromium Controller™ platform. Granulocytic (G-), Monocytic (M-), and Early (E-) MDSCs were identified as previously described by Bronte et al. (18)): G-MDSCs (Lin^-^ CD33^+^ CD11b^+^ CD14^-^ and CD15^+^ or CD66b^+^); M-MDSCs (Lin^-^ HLADR^low/-^ CD33^+^ CD11b^+^ CD14^+^ CD15^-^ CD66b^-^); and, E-MDSCs (Lin^-^ HLADR^low/-^ CD33^+^ CD11b^+^ CD14^-^ CD15^-^ CD66b^-^). T lymphocytes were identified by known gene markers: CD4^+^ T (*CD3D, CD4, CCR7*), CD8^+^ T (*CD3D, CD8A, CCR7*), activated CD4^+^ T (*CD3D, IL7R, CD4*), activated CD8^+^ T (*CD3D, CD8A, CCL5*), Treg (*CD3D, FOXP3, IL2RA*), and Th17 (*CD4, IL17*). Activated macrophages, monocytes, dendritic and plasmacytoid cells were labeled using additional oligo-tagged antibodies to CD3, CD127, CD16, CD183, CD4, CD196, CD25, and CD56. B cells were identified using CD19. Natural Killer (NK) and NK T-lymphocytes were identified using CD16 (*FCGR3A*), CD56 (*NCAM1*), and CD3 (*CD3G*). Complementary DNA (cDNA) libraries were constructed to assess gene expression (RNA) and surface phenotype (protein) for subpopulations simultaneously. Cell clusters were manually annotated based on their expression of known marker genes and leveraging both the RNA expression counts and antibody derived tag counts. Details of this analysis are provided in **Supplementary Data Sheet 1**.

### Processing of Sequencing Reads and Generation of Gene-Barcode Matrices

Raw sequencing reads were processed using Cell Ranger v3.0.0 to create a raw (unfiltered) gene-barcode matrix. Briefly, Cell Ranger mkfastq was used to make fastq files from bcl files. Next, Cell Ranger count was used for aligning sequencing reads to the hg19 reference genome (refdata-cellranger-hg19-3.0.0), obtained from https://support.10xgenomics.com/single-cell-gene-expression/software/release-notes/build using STAR. For confidently mapped reads, UMI sequences were collapsed and the number of UMI reads per gene were stored in the raw gene-barcode matrix (https://support.10xgenomics.com/single-cell-gene-expression/software/pipelines/latest/algorithms/overview).

### Filtering of Barcodes/Quality Control

We distinguished true cells from background droplets using the emptyDrops method implemented in the DropletUtils Bioconductor R package (19). By testing each cell *versus* an ambient RNA distribution, barcodes with a false discovery rate adjusted p-value < 0.01 were retained for further consideration. We performed a second quality control step to identify cells with low RNA content, possible doublets, or dead/damaged cells, in which we filtered cells based on the total number of UMIs per cell, the number of genes expressed, and the percentage of mitochondrial reads per cell. We used the scater R package to identify outlier identify cells in any of these metrics, where outliers were defined as three median absolute deviations (MADs) from the median (20). The scRNA-seq data were normalized using the NormalizeData function in the Seurat R package v 3.1.5 (21) in which the total counts for each cell were scaled to have 10000 total counts. The antibody counts were normalized using the same function with the centered log ratio transformation method.

### Dataset Integration and Dimensionality Reduction

The datasets were integrated as detailed by McCarthy et al. (20). Briefly, canonical correlation analysis (CCA) was performed to identify shared sources of variation across the datasets, and mutual nearest neighbors in the CCA space were identified to produce anchors between datasets. Highly variable genes accounting for the majority of the heterogeneity within each sample were identified with the FindVariableFeatures function in the Seurat R package v 3.1.5 (21), which fits a local polynomial regression (loess) model to the mean-variance relationship and selects the top 2000 genes with the greatest standardized deviation from the fitted model. Using these features, anchors between the datasets which correspond to similar cells across datasets were identified using the FindIntegrationAnchors function, and this was used as input into the IntegrateData function to generate an integrated dataset. For dimensionality reduction, expression values for each gene in the integrated dataset were scaled to have a mean of zero and standard deviation of one using the ScaleData function. Principal component analysis (PCA) was run on this matrix using the RunPCA function in Seurat. For visualization Uniform Manifold Approximation and Projection (UMAP), a common dimensionality reduction method in scRNA-seq (21), plots were created based on the top 30 principal components using the RunUMAP function in Seurat.

### Cell Cluster Differential Expression Analysis

Cell clusters were manually annotated based on their expression of known marker genes. Marker genes for each cluster were identified by comparing each individual cluster with the remaining pooled clusters for each sample using the Wilcoxon rank sum test implemented in the Seurat R package. Differentially expressed genes across conditions was done by pooling cells across subjects for each cell cluster using the Wilcoxon rank sum test. P-values were adjusted for multiple testing using the Bonferroni method. All analyses were performed using R version 3.6.3.

### Pathway Analysis of Non-Myeloid Cells

Differentially expressed genes for each cell of interest were functionally annotated using the R package clusterProfiler (22). The Kyoto Encyclopedia of Genes and Genomes (KEGG) and Gene Ontology (GO) databases were used to determine association with particular diseases and biological processes. For KEGG, the enrichment P-value cutoff was set to pvalueCutoff = 0.05 and for GO we set qvalueCutoff = 0.01.

## Results

Three of the four septic patients developed CCI and were sampled during that period. One of the septic patients had recovered sufficiently to be defined as a rapid recovery patient, although the patient was still hospitalized at the time of blood sampling. Two of the four patients with sepsis had fungal sepsis while the other two had bacterial sepsis. The mean age for the septic patients was 65 years; 75% were male and they were all studied post-sepsis day 14-21. Five healthy controls (mean age 42 years; 100% male) were also studied. Patient characteristics are detailed in **Supplementary Table 1**.

### Identification of Lymphoid/Non-Myeloid Leukocyte Cell Types

**Figure 1:**
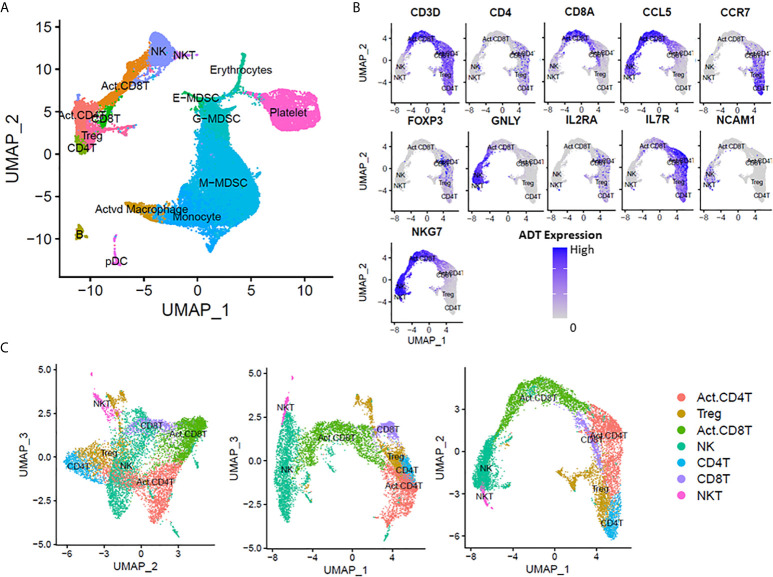
scRNA-seq analysis at 14-21 days post-sepsis *versus* healthy control. Cells depicted are from all subjects in the study, in each corresponding group (sepsis n=4, healthy n=5). Using Seurat’s method of integrating data across conditions/batches, the integration allows for joint clustering and to identify shared (or possibly unshared) cell clusters. Cells are visualized on uniform manifold approximation and projection (UMAP) plots colored by cell types. **(A)** UMAP representation of cell clusters identified in healthy patients *versus* late sepsis. **(B)** Annotation of T-lymphocyte subsets was performed manually using expression of *CD3D, CD4, CD8A, CCL5, CCR7, FOXP3, GNLY, IL2RA, IL7R, NCAM1* and *NKG7*. **(C)** UMAP representation of T-cell subset clusters from manual annotation identified in healthy patients *versus* late sepsis in three dimensions. (E-MDSC, early myeloid derived suppressor cell; G-MDSC, granulocytic myeloid derived suppressor cell; M-MDSC, monocytic myeloid derived suppressor cell; pDC, plasmacytoid dendritic cells).

We leveraged CITE-seq data to annotate cell clusters based on known cell markers (**Figure 1A**) as described in the Methods. We successfully identified 11 non-myeloid cell types: six T-lymphocyte subsets (naive CD4^+^, naive CD8^+^ activated CD4^+^, activated CD8^+^, NK T cells, and regulatory T-lymphocytes) (**Figures 1B, C**), NK cells, B-cells, and plasmacytoid dendritic cells (pDCs). Surprisingly, we were unable to positively identify any Th17 cells in our analysis (*via* expression of *IL17*). Th17 cells are known to be important for intact immunity, and their dysfunction can be associated with worse outcomes after sepsis (23, 24).

### Non-Myeloid Cells in Healthy *vs* Late Sepsis

#### T-Lymphocytes

In the analysis of healthy controls (n=5) with all sepsis patients day 14-21 (n=4), scRNA-seq revealed differential expression of 11 genes in CD4^+^, 30 genes in CD8^+^, 26 genes in activated CD4^+^, 50 genes in activated CD8^+^, and 32 genes in regulatory T (Tregs) lymphocytes (adjusted p-value < 0.01; **Supplementary Data Sheet 2** and **Supplementary Table 2**).

*IL32* was significantly upregulated in all T-lymphocyte subsets. The IL-32 family shares no known homology to other cytokines and is a pro-inflammatory cytokine that stimulates the secretion of other proinflammatory cytokines and chemokines *via* nuclear factor kB (NFkB) and p38 mitogen-activated protein kinase (MAPK) pathways (25, 26). In addition, T-lymphocytes and Tregs from septic patients did not have many differentially expressed genes previously reported to be directly immunosuppressive; however, there was upregulation of pro-inflammatory genes such as *STAT1, NEAT1, IL32, PSME1/2, CCL4* (MIP1β)*, and LIME1*.

*TNFSF10* (TRAIL) and *S100A8/9*, were found to be significantly upregulated in late sepsis CD4^+^ and CD8^+^ T- lymphocyte subsets, respectively. *S100A8/A9* encode for proteins that have many pro- *and* anti-inflammatory immune functions (27). *TNFSF10* encodes for a cytokine in the tumor necrosis factor family that mediates cell apoptosis in response to tumor cells and infection (28, 29). *STAT1* and *SETD2* are also both over-expressed in late sepsis CD4^+^ T-lymphocytes. SETD2 is known to directly mediate methylation of STAT1 and reinforces interferon-α Activated transcription of STAT1 in antiviral immunity (30).

Activated CD8^+^ T-lymphocytes also showed upregulation of MHC II genes such as *CD74, HLA-DRA, HLA-DRB1, HLA-DRB5, HLA-DQA1.* MHC II gene expression in T-lymphocytes has been described as a late marker of activation, associated with persistent inflammation in many autoimmune disorders (31–33). Not surprisingly, late sepsis CD4^+^ T-lymphocytes did not have differential expression of any HLA genes. Interestingly, late sepsis activated CD4^+^ T cells had high expression of *ETS1*, which has been implicated as a negative regulator of Th17 differentiation (34). This could partially explain the lack of Th17 cells seen in this analysis. Activated CD4^+^ and CD8^+^ T-lymphocytes also shared upregulation of genes *IRF1*, implicated in T-lymphocyte differentiation (35, 36).

Activated CD8^+^ T-lymphocytes from late sepsis had the most differentially expressed genes compared to the other T-lymphocyte subsets. *CD27*, which is important in the generation of T-lymphocyte memory and viral immunity, was specifically downregulated in late sepsis activated CD8^+^ T-lymphocytes (37). Late sepsis activated CD8^+^ T-lymphocytes also showed upregulation of cytotoxic genes *ITGB1, GZMA, GZMH, NKG7* (38–42). Yet, in late sepsis there was downregulation of other cytotoxic lymphocyte genes *KLRC3* and *KLRC1*. Interestingly, late sepsis activated CD4^+^ T-lymphocytes also demonstrated upregulation of some cytotoxic genes such as *GZMA, CCL5*, and *CST7* (40, 42, 43).

We utilized functional enrichment analysis to gain greater biological insight into the functional processes activated or inhibited in late sepsis (**Supplementary Table 3**). KEGG enrichment of differentially expressed genes in CD4^+^ and CD8^+^ T cells identified only one significant pathway (Ribosome). KEGG enrichment of differentially expressed genes in activated CD4^+^ T cells and activated CD8^+^ T cells identified three (ribosome, influenza A, and NOD-like receptor signaling) and 32 pathways, respectively (adjusted p-value < 0.05). Differentially expressed genes from activated CD8^+^ T cells in late sepsis were enriched for KEGG pathways associated with antigen processing and presentation, many viral diseases and autoimmune disorders.

Most of the differentially expressed genes found in each T-lymphocyte subset are genes important for cell function such as ribosomal, mitochondrial, glycolytic, transcription and binding proteins. The majority of these regulatory genes, especially ribosomal genes, are downregulated in late sepsis T-lymphocytes. This suggests a relative cell “exhaustion or dysfunction” in late sepsis, potentially playing a role in T-lymphocyte exhaustion known to occur after sepsis (44, 45). KEGG enrichment analysis showed significant overlap (adjusted p-value < 0.05) in the Ribosome pathway for all T-lymphocyte subsets. GO analysis of the differentially expressed genes illustrated transcriptome involvement of biological processes related to ribosomal function in all T-lymphocyte subsets, except activated CD4^+^ T cells, further supporting T-lymphocyte exhaustion in late sepsis (adjusted p-value <0.01; **Supplementary Table 3**).

#### Cytotoxic Lymphocytes (NK and NKT)

In the comparison between healthy subjects (n=5) and those with late sepsis (n=4), scRNA-seq revealed differential expression of 8 genes in NK T cells and 28 genes in NK cells (adjusted p-value < 0.01; **Supplementary Data Sheet 2** and **Supplementary Table 2**). NK T cells had differential downregulation mainly of cell regulatory genes. However, there was also downregulation of the cytotoxic lymphocyte gene *KLRC3*. Interestingly, NK cells also exhibited downregulation of the cytotoxic gene *KLRC3* along with downregulation of *KLRC1* and *KLRG1*. *KLRC3* encodes for an activating receptor, while *KLRC1* and *KLRG1* encode for inhibitory receptors (46). Downregulation of these genes suggest compromised NK cell and NK T cell function in late sepsis.

Significant terms from KEGG and GO enrichment analyses can be found in **Supplementary Table 3**. KEGG enrichment of the differentially expressed genes in NK cells showed overlap in many immune related pathways including antigen processing and presentation and natural killer cell mediated cytotoxicity. GO analysis of the differentially expressed genes in NK cells revealed overlap with significant biological processes such as antigen processing and presentation, antigen receptor mediated-signaling pathway, and cytokine signaling. Interestingly, differentially expressed genes in both NK and NKT cells enriched for multiple GO biological process terms related to protein targeting to the membrane. NKT cell genes also enriched for multiple GO biological processes pathways important for proper cellular function (ATP synthesis, ribosome biogenesis, rRNA processing, mitochondrial ATP synthesis. These findings further support NK cell and NK T cell dysfunction in late sepsis.

#### Plasmacytoid Dendritic Cells

There was differential expression of 16 genes in pDCs in the analysis of healthy controls (n=5) compared with all late sepsis patients (n=4) (adjusted p-value <0.01; **Supplementary Data Sheet 2** and **Supplementary Table 2**). There were only 4 upregulated genes, three of which are proinflammatory markers *S100A8, S100A12* and *FAM26F*. *S100A8* and *S100A12* are danger-associated molecular proteins (DAMPs) which are potent activators of innate immunity (47, 48). *FAM26F* encodes for a membrane glycoprotein which is implicated in immune cell-cell interactions. The synergistic expression of FAM26F is necessary for NK cell activation by dendritic cells (49, 50). Most of the differentially expressed genes downregulated in the late sepsis were important for cell function such as ribosomal, mitochondrial, glycolytic, transcription and binding proteins. One downregulated gene, *DNASE1L3*, which encodes for an endonuclease, has been demonstrated to be necessary for inflammasome-mediated cytokine secretion (51, 52). Enrichment analysis was significant for five KEGG terms (adjusted p-value < 0.05) and 30 GO terms (adjusted p-value <0.01). The significant GO terms include many pathways important for immune cell function, such as protein targeting to the membrane, organelle organization, regulation of inflammatory response and granulocyte chemotaxis (**Supplementary Table 3**). Together these genes highlight immunometabolic dysfunction and suggest pDCs as an important contributor to the persistent inflammation seen in chronic sepsis.

#### B-Cells

In the analysis of healthy controls (n=5) compared with all late sepsis patients (n=4), there were only 11 differentially expressed genes (adjusted p-value <0.01; **Supplementary Data Sheet 2** and **Supplementary Table 2**). The three downregulated genes encoded for ribosomal and mitochondrial proteins, suggesting immunometabolic dysfunction. Of the upregulated genes, there were 3 pro-inflammatory genes (*FAM26F, IFITM1, and AIM2*) all of which are inducible by interferon and important in the activation of the innate immune response (49, 53, 54). IFITM1 protein has also been reported to play a role in the inhibition of B cell line proliferation (54). AIM2 is an important cytoplasmic sensor that recognizes dsDNA for a wide range of pathogens, as well as host DNA, that triggers the release of many cytokines upon activation (53). This is not reflected in the enrichment analysis (**Supplementary Table 3**). There was significant overlap in only two GO biologic process pathways for protein oligomerization (adjusted p-value <0.01) and no significant KEGG pathways (adjusted p-value <0.05).

### Lymphocytes in Chronic Critical Illness

Within our sepsis cohort, three patients were classified as CCI and one patient as rapid recovery. Although our sample size for rapid recovery patients is too low for a direct analysis, there were enough CCI patients to determine if there are differences more specific to CCI patients compared to our previous analysis of all patients. Therefore, further analysis was performed to elucidate transcriptomic patterns in the lymphocytic cell types of late sepsis CCI patients compared to healthy controls. In the analysis of healthy controls (n=5) to late sepsis CCI patients (n=3), scRNA-seq revealed differential expression of 16 genes in CD4^+^, 30 genes in CD8^+^, 26 genes in activated CD4^+^, 52 genes in activated CD8^+^, and 37 genes in regulatory T (Tregs) lymphocytes. There was differential expression of 8 genes in NKT, 35 genes in NK, 11 genes in B, and 15 genes in pDC (adjusted p-value < 0.01; **Supplementary Data Sheet 3** and **Supplementary Table 4**). The lower number of differentially expressed genes, as well as, the high number of common genes between analyses suggests that the CD8^+^ T, NK T cells, pDCs, and B cells in late sepsis have begun trending back towards homeostasis, regardless of clinical trajectory. Of note, the CD8^+^ T-, NKT- and B-lymphocytes differentially expressed genes from the analysis of all late sepsis patients are the same as the differentially expressed genes in the analysis for CCI. The differentially expressed genes for the other non-myeloid cells do not differ wildly between the two analyses, but there are some differences worth mentioning.

As hypothesized, scRNA-seq analysis revealed differentially expressed genes important for both immunosuppression and inflammation in all CCI T-lymphocyte subsets, just as reported for all sepsis patients above. The sub analysis of healthy *versus* CCI CD4^+^ T-lymphocytes revealed unique differential expression of five genes not seen in the analysis of all late sepsis patients (upregulated: *HBB, ETS1*; downregulated: *SMDT1, RPL41, MTRNR2L12*). There was also unique expression of three genes in CCI activated CD4^+^ T-lymphocytes (upregulated: *AL592183.1*; downregulated: *PLP2, MTRNR2L12*), five genes in activated CD8^+^ T-lymphocytes (upregulated: *MT-ND6, THEMIS*; downregulated: *RPL38, RPL41, MTRNR2L12*); ten genes in Tregs (upregulated: *SORL1, ETS1, IRF1, EIF1AX, DDX5, SMCHD1, LINC00657, USP22, tNSF10*; downregulated: *MTRNR2L12*); and eight genes in NK cells (upregulated: *S100A8, CD74, HLA-DPA1, JUNB, MT-ND6, DUSP, AL592183.1*; downregulated: *MTRNR2L12)*. Most of the uniquely expressed genes in CCI lymphocytes encoded for regulatory proteins – mitochondrial, ribosomal and transcriptional However, Tregs also saw an increased expression level of proinflammatory genes *IRF1* and *TNSF10.* While there were no differences noted in this analysis of CCI NKT cells compared to analysis of all sepsis NKT cells, NK cells in CCI had a unique upregulation of inflammatory genes *S100A8, CD74, JUNB*, and *DUSP1*. Although differential regulatory function is reflected in the enrichment terms (**Supplementary Table 5**) which are similar to the previous analysis of all sepsis patients compared to healthy controls, there are no unique pathways to suggest a concerted increase of inflammation in non-myeloid populations in CCI late sepsis. However, this differential gene expression in conjunction with the lack of significant overlap in related pathways, could represent a dysregulated attempt to increase inflammation in CCI late sepsis when compared to RAP late sepsis and healthy controls. This data also suggests that the persistent inflammation seen in PICS is not driven by non-myeloid cells.

### Lymphocytes After Bacteremia *Versus* Fungemia

Finally, we compared scRNA-seq profiles of late sepsis lymphocytes based on initial septic insult (bacterial *vs* fungal). Gene expression analysis between the late sepsis patients with bacteremia (n=2) *versus* fungemia (n=2) revealed differential expression of 25 genes in CD4^+^ T-lymphocytes, 44 genes in CD8^+^ T-lymphocytes, 93 genes in activated CD4^+^ T-lymphocytes, 368 genes in activated CD8^+^ T-lymphocytes, 24 genes in Tregs, 9 genes in NK T cells, 249 genes in NK cells, 16 genes in B-cells, and ten genes in pDCs (adjusted p-value< 0.01). However, many of these genes differed only modestly in expression; thus, we applied a further cutoff value of log fold-change (FC) > │0.4│ (corresponding to standard FC > │1.5│) to select significant genes. Gene analysis with the more stringent criteria (adjusted p-value < 0.01 and log FC > │0.4│) revealed differential expression of 19 genes in CD4^+^ T-lymphocytes, 44 genes in CD8^+^ T-lymphocytes, 41 genes in activated CD4^+^ T-lymphocytes, 161 genes in activated CD8^+^ T-lymphocytes, 21 genes in Tregs, 9 genes in NK T-cells, 89 genes in NK cells, 11 genes in B-cells, and six genes in pDCs (**Supplementary Data Sheet 4** and **Supplementary Table 6**).

Interestingly, the naive and activated CD8^+^ T-lymphocytes in bacterial sepsis demonstrated a more cytotoxic-like pattern with upregulation of cytotoxic genes such as *GZMA, GZMH, GZMK, CCL5, CST7*, and *NKG7* (38, 40–42). Activated T-lymphocytes from late bacterial sepsis showed upregulation of many genes for DAMPs and HLA proteins. B cells and pDCs of late sepsis showed differential expression mainly of genes for ribosomal, mitochondrial, and transcription proteins. Of note, all lymphocytes in late bacterial sepsis showed increased expression of adult hemoglobin genes *HBB* ± *HBA2* when compared to those lymphocytes in fungal sepsis. Although the function of *HBB* and *HBA2* are unknown in lymphocytes, some studies have shown increased expression in non-erythroid cells to be associated with hypoxia (55). Thus, it is possible that the expression of hemoglobin genes may reflect differences in the sepsis phenotype (Skin/Soft Tissue *vs* Vascular/Bloodstream) rather than initial microbial insult.

Enrichment analysis results can be found in **Supplementary Table 7**. Supporting our claim above, activated CD8^+^ T differential genes enriched for terms that support a more cytotoxic-like pattern natural killer cell mediated cytotoxicity, phagosome, and proteasome pathways. Enrichment of the differential genes in all non-myeloid subsets in late bacterial *versus* late fungal sepsis demonstrated significant overlap in many immune cell related pathways such as antigen processing and presentation, chemokine signaling, NK-kappa B signaling, granulocyte chemotaxis, and immune cell differentiation.

## Discussion

Historically, 30-day mortality has been the primary outcome of interest in surgical sepsis populations (56–58). However, many sepsis patients survive thanks to earlier identification and improved subsequent therapy (3, 4). Thus, sepsis research is adapting to evaluate long-term outcomes, such as functional status, cognitive status, and one-year morality, as primary outcomes (3, 4, 57, 59, 60). Ongoing research suggests that the dismal long-term outcomes experienced by CCI patients are due to a dysregulated immune response that fails to return to homeostasis (5, 6, 9, 11, 13), which we have identified as the PICS endotype (12).

This study utilized scRNAseq to reveal novel gene expression patterns in circulating non-myeloid cells of late sepsis. Similar to previous studies, our study supports the hypothesis that immune dysregulation and failure to restore immune homeostasis is associated with the development of CCI among sepsis survivors. As noted in other sepsis cohorts, there is substantial variability in transcriptional changes in inflammatory genes, both early and late after sepsis, for CCI and rapid recovery patients (61–64). Neither early nor late sepsis transcriptomic patterns identify a distinctive pro-inflammatory or immunosuppressive phase (65). Rather, there is an aberrance of both inflammatory and immunosuppressive genes simultaneously, suggesting global immune dysregulation further promoting PICS as the underlying pathobiology of late sepsis.

Our study highlights that lymphoid populations have a transcriptomic profile more suggestive of immune and metabolic dysfunction than inflammation at 14-21 days post-sepsis. T-lymphocytes had some overexpression of both pro-inflammatory and immunosuppressive genes in late sepsis, but not to the levels seen in myeloid populations (12). In contrast, both pDCs and platelets appear to play an important role in the persistent inflammation seen in chronic sepsis with upregulation of genes for pro-inflammatory cytokines and chemokines. Particularly in CCI late sepsis, transcriptomic patterns suggest the potential for persistent inflammation *via* NK cells and Treg lymphocytes. The transcriptomic profiles could represent a compensatory upregulation of these inflammatory genes to combat the relative immunosuppression. Importantly, our analysis also suggests that the transcriptomic profile may be influenced not only by initial microbial insult and clinical trajectory but also by sepsis endotype/initial site of infection.

Surprisingly, we were unable to identify circulating Th17 lymphocytes in our study population. Specifically, we were unable to identify the expression of *IL17* messenger RNA (mRNA) in any of our T-lymphocyte subsets. Th17 lymphocytes are one of the effector cell types differentiated from activated CD4+T- lymphocytes *via* induction from TGF-β, IL-6, IL-21 (24, 66). Th17 cells have been demonstrated to be crucial in neutrophil recruitment for defense against extracellular pathogens and crucial in mucosal host defense (67). Th17 lymphocytes play an important role in autoimmune diseases, promoting chronic inflammation, but this has not been described in late sepsis. In one study, circulatory Th17 lymphocyte counts were higher in survivors with severe sepsis than in non-survivors and counts were increased after 6 days in severe sepsis, suggesting an association of Th17 with severe sepsis survival (68). To our knowledge, this is the first study to look at T-lymphocytic subsets after 7 days post-sepsis. Our study suggests that there is a significant decrease in circulating Th17 cells at 14 days post sepsis, which could indicate decreased differentiation of Th17 lymphocytes. Importantly, Th17 cells are the major producers of IL17 cytokine, which has been documented to increase extramedullary hematopoiesis and bone marrow granulopoiesis (67, 69–71). Therefore, the lack of Th17 lymphocytes in late sepsis/CCI may play a critical role in the persistent dysfunctional hematopoiesis of PICS.

We also observed that the majority of the downregulated differentially expressed genes in non-myeloid cells of late sepsis encode for glycolytic, ribosomal and mitochondrial proteins. Previous studies report similar alterations in leukocyte transcriptomic profiles in early inflammation (72, 73). However, the perseverance of this transcriptome has not been well delineated in late sepsis. The functional consequences of downregulation of these genes in the T-lymphocyte subsets are consistent with what has been described as “T-lymphocyte exhaustion”, which is characterized by dysfunctional differentiation and decreased immune response to a new inflammatory stimulus, usually to chronic viral infections (74, 75). The repression of genes encoding for ribosomal and mitochondrial proteins has been associated with a decrease in T-lymphocyte function and differentiation (76, 77). This could explain our failure to confidently identify Th17-lymphocytes in these late sepsis patients. The lack of definitive Th17 cells could contribute to the increase susceptibility to secondary infections in CCI sepsis patients (78, 79).

The main limitation of this study was that it was performed in a limited number of surgical sepsis patients. Further validation of the differentially expressed genes with more patients, especially those that rapidly recovered, is warranted. Additionally, we were only able to analyze immune cell transcriptome patterns at one time point in late sepsis. Future transcriptomic analysis with more time points is warranted to analyze time-dependent genomic expression patterns of late sepsis in sepsis survivors (14). Finally, this is a descriptive study. It is important to note that mRNA expression does not always correlate with protein expression due to many epigenetic and posttranslational factors. We recognize that the KEGG and GO enrichment analyses did not perfectly align with how we interpret gene interaction within each non-myeloid cell. Yet this does not trivialize the importance of our findings. In fact, this highlights our lack of understanding of the immune cell transcriptomic response and subsequent endotype in late sepsis. Further functional studies to elucidate the specific role of these differentially expressed genes in chronic sepsis are warranted. In addition, the circulating leukocyte transcriptome does not necessarily represent the leukocyte transcriptome within individual tissues. However, the work presented in this manuscript highlights a number of genes and non-myeloid cell subsets that could be targeted for therapy in those sepsis patients that have CCI and adverse outcomes as a consequence of PICS.

## Conclusions

Utilizing scRNA-seq, we identified unique transcriptomic profiles of lymphocytic cell types in late sepsis patients. scRNA-seq analysis demonstrated that circulating lymphocytic cells maintain a transcriptomic profile that is predominantly immunosuppressive with low-grade pro-inflammatory characteristics in late sepsis patients. In addition, the type of infecting organism (e.g. bacterial *vs* fungal) can alter the lymphocyte transcriptome later in sepsis, further highlighting the need for personalized immunotherapy. Importantly, this data highlight that the persistent global immune dysfunction in late sepsis extends to nearly all lymphocytic subsets which are all likely playing a role in the increased secondary infections, increased late mortality, and poorer prognosis seen in CCI. Future studies with particular attention to Th17 lymphocytic dysfunction may reveal this subset as a major target to reflect poor prognosis and adverse outcomes in late sepsis. However, many more studies are warranted to completely understand the PICS endotype in order to devise an effective treatment plan for CCI patients in late sepsis.

## Data Availability Statement

The datasets presented in this study can be found in online repositories. The names of the repository/repositories and accession number(s) can be found below: https://www.ncbi.nlm.nih.gov/geo/query/acc.cgi?acc=GSE175453, GSE175453.

## Ethics Statement

The studies involving human participants were reviewed and approved by University of Florida’s Institutional Review Board. The patients/participants provided their written informed consent to participate in this study.

## Author Contributions

Project conception and design: DD, RB, MB, XD, MD, RU, DN, JR, TB, AB, FM, LM, AM, and PE. Data collection: MB, MD, RU, DN, and JR. Data analysis and interpretation: DD, XD, RB, and PE. Original manuscript drafting: DB, RB, XD, and PE. Manuscript review and editing: DD, RB, MB, XD, MD, RU, DN, JR, TB, AB, FM, LM, TJ, AM, and PE. Manuscript final approval: DD, FM, LM, TL, AM, and PE. All authors contributed to the article and approved the submitted version.

## Funding

This work was supported in part by the following National Institutes of Health grants: NIGMS: R01 GM-104481, R01 GM-113945, P50 GM-111152, RM1 GM-139690-01. T32 GM-008721; and NIA: 1P30AG028740.

## Conflict of Interest

The authors declare that the research was conducted in the absence of any commercial or financial relationships that could be construed as a potential conflict of interest.

## Publisher’s Note

All claims expressed in this article are solely those of the authors and do not necessarily represent those of their affiliated organizations, or those of the publisher, the editors and the reviewers. Any product that may be evaluated in this article, or claim that may be made by its manufacturer, is not guaranteed or endorsed by the publisher.

## Supplementary Material

Supplementary Data Sheet 1 Summary of cell cluster annotation.

Supplementary Data Sheet 2 Volcano plots for each cell cluster of the top up and down regulated genes between healthy subjects *versus* all late sepsis non-myeloid cells. Each dot represents a gene statistically enriched or reduced within the cell cluster. The volcano plot compares natural log fold-change (healthy mean divided by sepsis mean; x-axis) with adjusted p-values (y-axis). Significance of differential gene expression was determined with adjusted p-value (p adj.) < 0.01.

Supplementary Data Sheet 3 Volcano plots for each cell cluster of the top up and down regulated genes between healthy subjects *versus* late sepsis chronic critical illness non-myeloid cells. Each dot represents a gene statistically enriched or reduced within the cell cluster. The volcano plot compares natural log fold-change (healthy mean divided by sepsis mean; x-axis) with adjusted p-values (y-axis). Significance of differential gene expression was determined with adjusted p-value (p adj.) < 0.01.

Supplementary Data Sheet 4 Volcano plots for each cell cluster of the top up and down regulated genes between bacterial *versus* fungal late sepsis non-myeloid cells. Each dot represents a gene statistically enriched or reduced within the cell cluster. The volcano plot compares natural log fold-change (healthy mean divided by sepsis mean; x-axis) with adjusted p-values (y-axis). Significance of differential gene expression was determined with adjusted p-value (p adj.) < 0.01 and │FC│>0.4.

Supplementary Table 1 Patient characteristics.

Supplementary Table 2 Significant differentially expressed genes by cell type in analysis of Healthy controls vs All Sepsis patients. Differentially expressed genes are listed for each cell type (individual tabs). Natural log fold-changes are calculated as healthy mean divided by sepsis mean, i.e. negative value of logFC indicates higher expression in sepsis patients. PCT represents the proportion of cells that the corresponding gene is detected.

Supplementary Table 3 Kyoto Encyclopedia of Genes and Genomes (KEGG) pathway and Gene Ontology (GO) biological process functions pathway analyses of differentially expressed genes in healthy *versus* all late sepsis non-myeloid cells.

Supplementary Table 4 Significant differentially expressed genes by cell type in analysis of Healthy controls vs Chronic Critical Illness Sepsis patients. Differentially expressed genes are listed for each cell type (individual tabs). Natural log fold-changes are calculated as healthy mean divided by sepsis mean, i.e. negative value of logFC indicates higher expression in sepsis patients. PCT represents the proportion of cells that the corresponding gene is detected.

Supplementary Table 5 Kyoto Encyclopedia of Genes and Genomes (KEGG) pathway and Gene Ontology (GO) biological process functions pathway analyses of differentially expressed genes in healthy *versus* chronic critical illness late sepsis non-myeloid cells.

Supplementary Table 6 Significant differentially expressed genes by cell type in analysis of Bacterial Sepsis vs Fungal Sepsis patients. Differentially expressed genes are listed for each cell type (individual tabs). Natural log fold-changes are calculated as healthy mean divided by sepsis mean, i.e. negative value of logFC indicates higher expression in sepsis patients. PCT represents the proportion of cells that the corresponding gene is detected.

Supplementary Table 7 Kyoto Encyclopedia of Genes and Genomes (KEGG) pathway and Gene Ontology (GO) biological process functions pathway analyses of differentially expressed genes in bacterial *versus* fungal late sepsis non-myeloid cells.
