# Supplementary material for: A Novel Single Cell RNA-seq Analysis of Non-Myeloid Circulating Cells in Late Sepsis
Source: Front Immunol. 2021 Aug 16;12:696536. doi: 10.3389/fimmu.2021.696536 (PMC8415415; doi:10.3389/fimmu.2021.696536)
Supplement: Supplementary Data Sheet 1 — Summary of cell cluster annotation. [file DataSheet_1.pdf]

Supplementary Content 1. Summary of Cell Cluster Annotation

We began with cluster annotation as previously described in Darden et al (2021). Following data integration of all 9 samples, we extracted the previous cell annotations from the Sepsis 1, Sepsis 2, Healthy 1, and Healthy 2 samples and mapped the additional five samples to the existing annotations based on their UMAP coordinates. Rather than re-annotating all cells, this was done as three of the five samples had failure in capturing the antibody tags (Sepsis 3 and Healthy samples 3&4). Using the two-dimensional UMAP embeddings, we used a knn classifier to assign cells from the new samples to the existing cluster annotations. This served as our base cluster annotation on all samples. Next, based on the gene expression across all samples, we further annotated clusters that we previously did not have enough cells to identify. We identified a cluster of erythrocytes based on expression of HBB and HBA2 (Supplementary Figure 1A). We also identified a cluster of B-cells based on the expression of CD19 (Supplementary Figure 1B). Next, we further refined the T-cell clusters into 7 groups: Treg, Act.CD4T, Act.CD8T, NKT, NK, CD4T, CD8T. These clusters were based on the gene expression of well-known marker genes: *CD3D, CD4, CD8A, CCL5, CCR7, FOXP3, GNLY, IL2RA, IL7R, NCAM1* and *NKG7*. First, we subset the base-level T-cell annotated cells and applied the shared nearest neighbor (SNN) algorithm using the FindClusters function in the Seurat package. This resulted in 17 total clusters. Clusters were considered duplicated if they had the exact same marker genes expressed and were merged. For clusters that expressed marker genes from multiple clusters, we assigned the cluster based on the proportion of zero counts. For example, if marker gene X of a cell type A and marker gene Y of a cell type B were both highly expressed in a cluster, but marker gene Y was more sparsely expressed (undetected) then the cluster was assigned as cell type A.

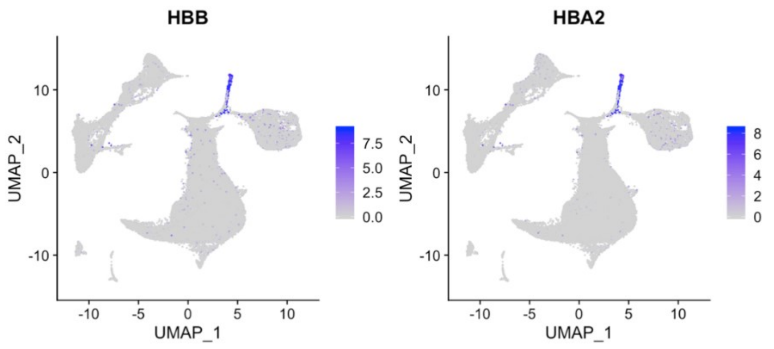

Supplementary Figure 1A. UMAP plot showing the gene expression of erythrocyte markers HBB and HBA2.

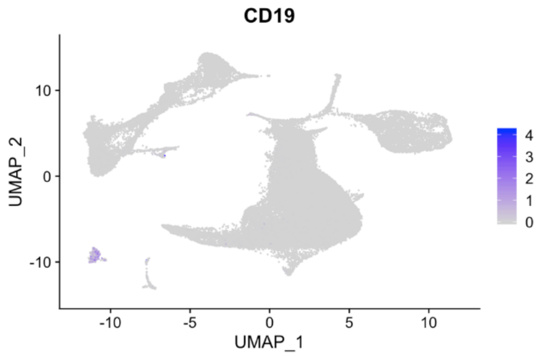

Supplementary Figure 1B. UMAP plot showing the gene expression of B-cell marker CD19.

References

Darden, Dijoia B., et al. "Single cell RNA-SEQ of human myeloid derived suppressor cells in late sepsis reveals multiple subsets with unique transcriptional responses: a pilot study." *Shock* (2021).
