# Supplementary material for: A Novel Single Cell RNA-seq Analysis of Non-Myeloid Circulating Cells in Late Sepsis
Source: Front Immunol. 2021 Aug 16;12:696536. doi: 10.3389/fimmu.2021.696536 (PMC8415415; doi:10.3389/fimmu.2021.696536)

Supplementary Data Sheet 2. Volcano plots for each cell cluster of the top up and down regulated genes between healthy subjects versus all late sepsis non-myeloid cells.

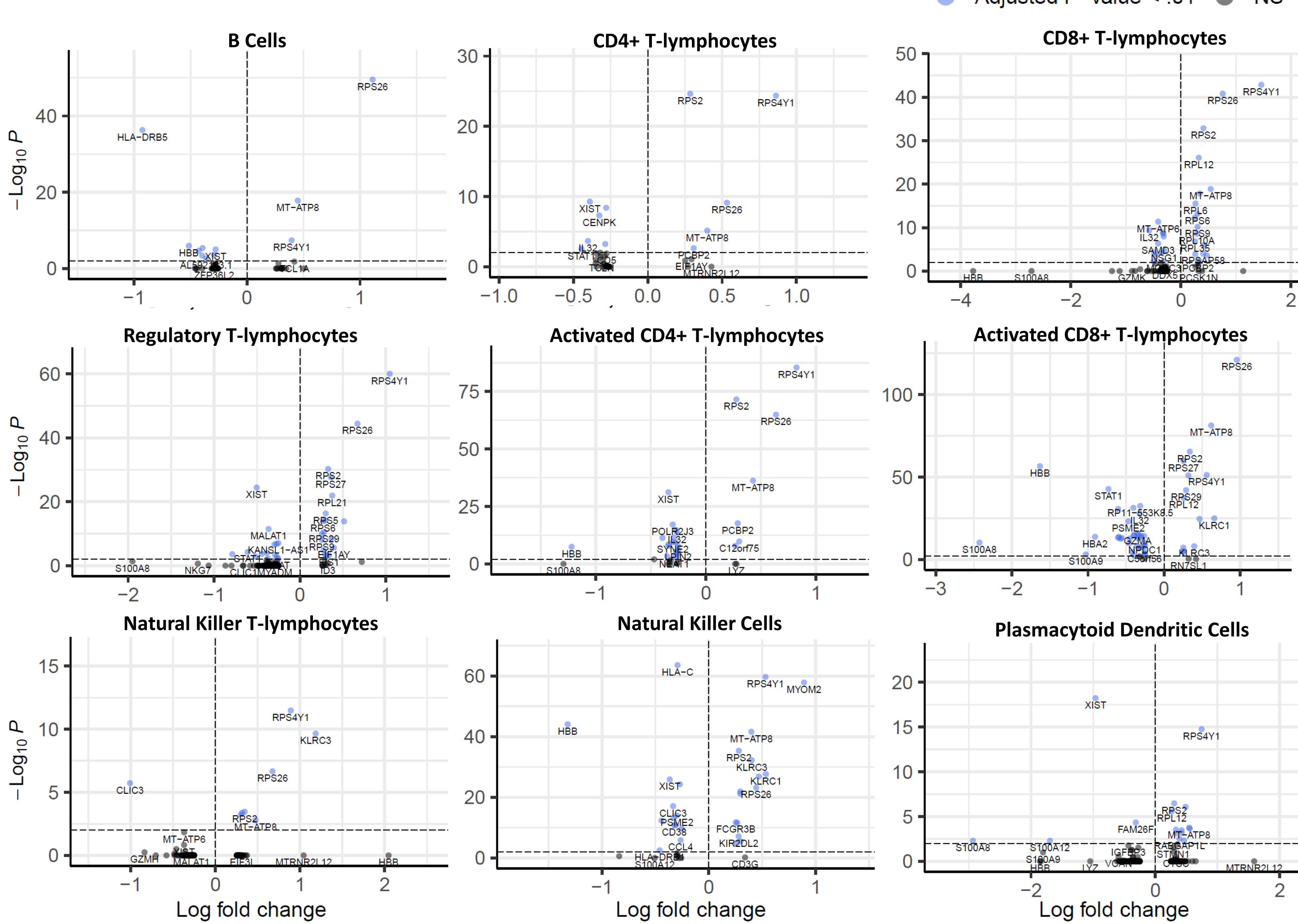

Supplement: Supplementary Data Sheet 2 — Volcano plots for each cell cluster of the top up and down regulated genes between healthy subjects versus all late sepsis non-myeloid cells. Each dot represents a gene statistically enriched or reduced within the cell cluster. The volcano plot compares natural log fold-change (healthy mean divided by sepsis mean; x-axis) with adjusted p-values (y-axis). Significance of differential gene expression was determined with adjusted p-value (p adj.) < 0.01. [file DataSheet_2.pdf]
